# Pregnant and breastfeeding women’s attitudes and fears regarding the COVID-19 vaccination

**DOI:** 10.1007/s00404-021-06297-z

**Published:** 2021-10-27

**Authors:** Nora K. Schaal, Janine Zöllkau, Philip Hepp, Tanja Fehm, Carsten Hagenbeck

**Affiliations:** 1 Department of Experimental Psychology, Heinrich-Heine-University Düsseldorf, Universitätsstraße 1, 40225 Düsseldorf, Germany; 2 Department of Obstetrics, University Hospital, Jena, Germany; 3 Clinic for Gynecology and Obstetrics, University Clinic, Augsburg, Germany; 4 Clinic for Gynecology and Obstetrics, HELIOS University Hospital Wuppertal, University Witten/Herdecke, Wuppertal, Germany; 5 Clinic for Gynecology and Obstetrics, University Clinic, Düsseldorf, Germany

**Keywords:** COVID-19, Vaccination, Pregnant, Breastfeeding, Anxiety

## Abstract

**Purpose:**

The COVID-19 vaccination is probably the most important source to fight the COVID-19 pandemic. However, recommendations and possibilities for vaccination for pregnant and breastfeeding women are inconsistent and dynamically changing.

**Methods:**

An anonymous, online, cross-sectional survey was conducted among pregnant and breastfeeding women in Germany between 30th March and 19th April 2021 addressing COVID-19 vaccination attitudes including the underlying reasons for their decision. Additionally, anxiety regarding a SARS-CoV-2 infection and a symptomatic course of the infection were evaluated.

**Results:**

In total, 2339 women (*n* = 1043 *pregnant* and *n* = 1296 *breastfeeding*) completed the survey. During pregnancy the majority (57.4%) are not in favour of receiving the vaccine, 28.8% are unsure and only 13.8% would get vaccinated at the time of the survey. In contrast, 47.2% would be in favour to receive the vaccine, if more scientific evidence on the safety of the vaccination during pregnancy would be available. Breastfeeding women show higher vaccination willingness (39.5% are in favour, 28.1% are unsure and 32.5% not in favour). The willingness to be vaccinated is significantly related to the women’s anxiety levels of getting infected and to develop disease symptoms. Main reasons for vaccination hesitancy are the women’s perception of limited vaccination-specific information, limited scientific evidence on vaccination safety and the fear to harm the fetus or infant.

**Conclusions:**

The results provide important implications for obstetrical care during the pandemic as well as for official recommendations und information strategies regarding the COVID-19 vaccination.

## Introduction

The COVID-19 pandemic with its accompanying restrictions is still dominating everyday life in most countries all over the world. Studies investigating the impact of COVID-19 infections in pregnancy have revealed that an infection is associated with increased risks of severe maternal and fetal complications (maternal admissions to intensive care units and risk for mechanical ventilation [1–4], comorbidities such as preeclampsia and thrombosis [5–8], maternal mortality [1, 2, 8], preterm births [2, 9], admission to the NICU [10] and stillbirths [9]). Vertical transmission of the virus to the baby is possible, but rare [11–13]. Regarding the course and outcome of the COVID-19 disease in pregnant women, it has been shown that a higher age (> 35 years), higher body mass index, hypertension and further comorbidities like diabetes are associated with a worse prognosis [4, 14], highlighting that the risk of a severe COVID-19 infection is especially high for women with these risk factors.

Thus, infection prevention in pregnant and breastfeeding women should be high priority. Available since December 2020, the COVID-19 vaccination is probably the most important source to protect health and lives in the fight against the COVID-19 pandemic. Up to now, medical approval was not given for pregnant or breastfeeding women in general. Recommendations and possibilities for vaccination in this vulnerable group are inconsistent across countries and dynamically changing. Prospective research investigating the effectiveness and safety of the COVID-19 vaccination in pregnant and breastfeeding women is missing as pregnant women are often excluded in clinical trials [15]. However, a recent cohort study investigating the safety of mRNA Covid-19 vaccines including 3.958 pregnant women revealed no obvious safety signals. Proportions of adverse pregnancy complications and neonatal outcomes (i.e., miscarriage, stillbirth, preterm birth, neonatal death) did not differ to pre-COVID-19 data [16]. Additionally, it has been shown that receiving COVID-19 mRNA vaccine was reliably immunogenic in pregnant women, and vaccine-elicited antibodies were transported to infant cord blood and breast milk [17]. Increasing evidence indicates that SARS-CoV-2-specific IgA and IgG antibodies are present in breast milk post-vaccination, suggesting that infants may also be better protected [18, 19].

In some countries, like the USA and Israel national recommendations for general vaccination of pregnant and breastfeeding women exist. The American College of Obstetricians and Gynecologists (ACOG) recommends that pregnant individuals have access to COVID-19 vaccines [20]. However, at the time of recruitment for the present study, the German national vaccination commission (*Ständige Impfkommission, STIKO*) does not recommend the vaccination for pregnant and breastfeeding women in general. But vaccination can be received after counseling through healthcare professional addressing benefits and risks of having the vaccine and reaching a joint decision based on individual circumstances.

The aim of the present study was to explore the attitude to COVID-19 vaccination of pregnant and breastfeeding women in Germany.

## Methods

An anonymous, online, cross-sectional survey was conducted between 30th March and 19th April 2021. All pregnant and breastfeeding women in Germany were eligible. Participants were recruited through midwifes, gynecologists and social media (i.e., Facebook, Instagram). The survey was administered through the platform soscisurvey.de [21]. Participants gave informed written consent at the beginning of the online survey. Time for completion was approximately 10 min.

During the time of recruitment approximately 10% of the German population were vaccinated, only people over 70 years of age and specific (risk) groups (i.e., hospital and medical staff, people with severe illnesses) were eligible to be vaccinated. The COVID-19 vaccination was officially not recommended for pregnant or breastfeeding women at the time of recruitment.

### Materials and procedure

A questionnaire was developed to evaluate the willingness to receive a COVID-19 vaccine during pregnancy or while breastfeeding and attitudes regarding the vaccine. Firstly, demographics (age, relationship status and highest qualification) were evaluated. Then, we asked the women whether they have preexisting health issues which classify them as high risk for severe COVID-19 infections (i.e., as COVID-19 risk patients) and about diagnosed COVID-19 infections in the past.

Subsequently, it was determined whether the women were pregnant or breastfeeding, which served as a filter for the following questions. Specific questions for each group (i.e., gestational age, risk pregnancy (yes/no), parity (primi/multi) for the *pregnancy* group and age of the child for the *breastfeeding* group) were administered. Then the same questions for both groups were evaluated covering the following topics: preexisting vaccinations against COVID-19, side effects after the vaccination and reasons for deciding to be vaccinated (multiple-select questions); willingness to be vaccinated if they would receive an offer now, reasons for and against a vaccination (multiple-select questions) and preferable vaccines (BioNTech; Moderna, AstraZeneca and Johnson and Johnson); which information regarding the COVID-19 vaccination and from whom they would find helpful (multiple-select questions); whether they would decide to be vaccinated when ongoing studies conducted in the USA, UK etc. would show that the COVID-19 vaccination is safe for pregnant women (i.e., the risks are comparable as for non-pregnant women). Additionally, we asked the women how anxious they are to get infected and how anxious they are to develop severe COVID-19 symptoms when infected. We asked them to indicate their anxiety levels regarding these two aspects (anxiety to be infected and anxiety regarding COVID-10 symptoms) by placing a cross on a 100 mm visual analogue scale ranging from *0* = *not at all anxious* to *100* = *very anxious*. We then measured the distance in mm from 0 with a ruler and higher scores indicate higher anxiety levels.

### Statistical analysis

The analysis was performed using IBM SPSS^®^ Version 27. Descriptive statistics are presented as *n* and % for categorical variables and means ± standard deviations for to continuous variables.

For the visual analogue scales for anxiety related to COVID-19 infection and severe COVID-19 symptoms mean scores were compared with within-subject *t*-tests for each group to investigate which aspect led to more anxiety. Additionally, we grouped the participants into low and highly anxious women. Women who indicated anxiety levels ≤ 50 were categorized as *low anxiety* group and values ≥ 51 were classified as the high anxiety group for infection and symptoms, respectively. Then, chi-square-tests were calculated to investigate whether the willingness to be vaccinated differs depending on (1) whether women indicated low or high anxiety to be infected and to develop severe symptoms respectively, (2) whether they are COVID-19 risk patients or not.

## Results

### Group characteristics

**Fig. 1:**
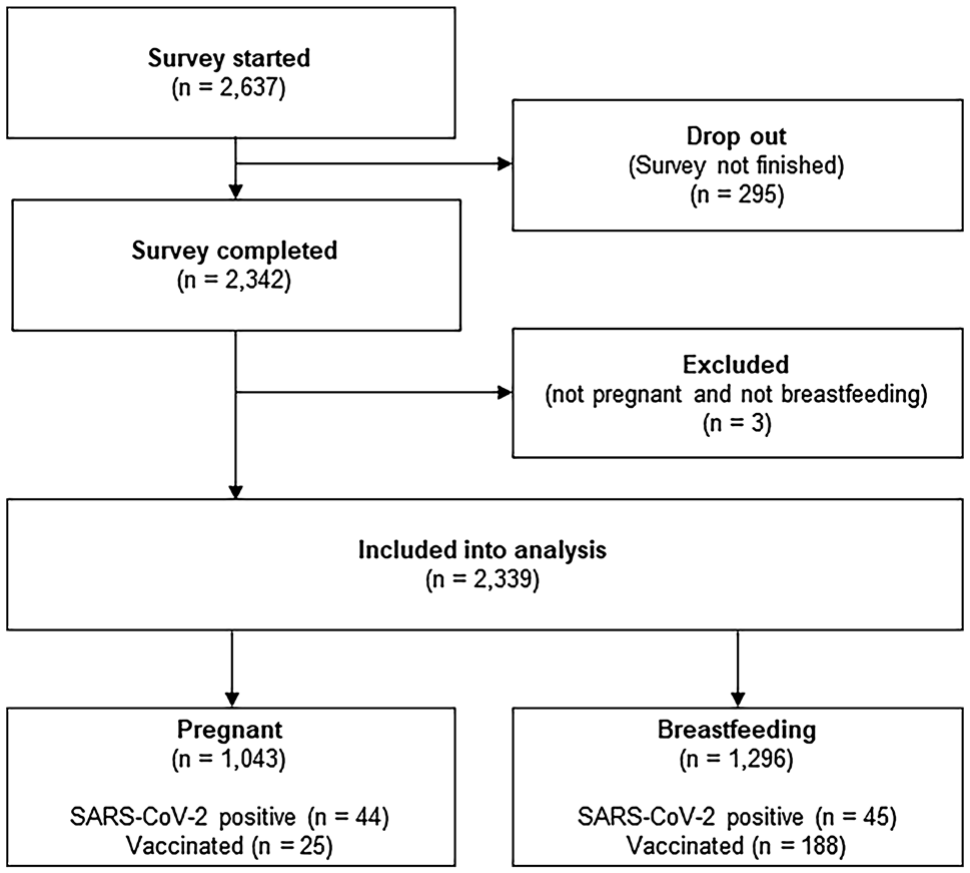
Flowchart of the study sample

Data from of 2339 women (1043 pregnant and 1296 breastfeeding) were included in the analysis. The study flow chart is presented in Fig. 1. Sample characteristics are presented in Table 1.

**Table 1:** Overview of sample characteristics

	Pregnant group	Breastfeeding group
*n*	1043	1296
Age (in years)	31.8 ± 4.3	32.4 ± 4.4
Gestational age (in weeks)	24.7 ± 9.1	–
Risk pregnancy	40.1% (*n* = 418)	–
Age breastfeeding child (in days)	–	328 ± 263
COVID-19 risk patients	20.1% (*n* = 201)	20.2% (*n* = 262)
In a relationship	98.4% (*n* = 1027)	98.1% (*n* = 1272)
Highest education		
Did not complete school	0.5% (*n* = 5)	0.1% (*n* = 1)
First school qualification	21.4% (*N* = 223)	18.3% (*n* = 236)
Second school qualification	25.4% (*n* = 264)	25.1% (*n* = 325)
University degree	46.7% (*n* = 487)	51.2% (*n* = 512)
PhD	4.6% (*n* = 48)	4.9% (*n* = 64)
No information	1.5% (*n* = 16)	0.5% (*n* = 6)

### Main results

#### Pregnant group

In the *pregnant group,* 4.2% (*n* = 44) indicated that they have been infected with SARS-CoV-2. Of these 44 women, 29 were already pregnant when tested positive and most of them only had mild symptoms (68.2%), while 13.6% had no symptoms and 18.2% severe symptoms.

Twenty-five (2.4%) have received the COVID-19 vaccination (13 received both doses and 12 the first doses). Of these 25 women, 13 were fully vaccinated during pregnancy, 8 received the first doses before pregnancy and will receive the second during pregnancy and 4 were vaccinated before pregnant. The majority (*n* = 16, 64.0%) received BioNTech/Pfizer, followed by AstraZeneca (*n* = 7) and Moderna (*n* = 2). Reported side effects were a sore arm where the needle went in (*n* = 20, 80%), feeling unwell generally (*n* = 11, 44%), fever (*n* = 5, 20%) and chills (*n* = 6, 24%).

The most common reasons for receiving the vaccination were because of their job (*n* = 22, 88.0%), because they were more anxious to be infected then about side effects of the vaccination (*n* = 13, 52%) and because they want to protect their unborn (*n* = 9, 36.0%).

**Fig. 2:**
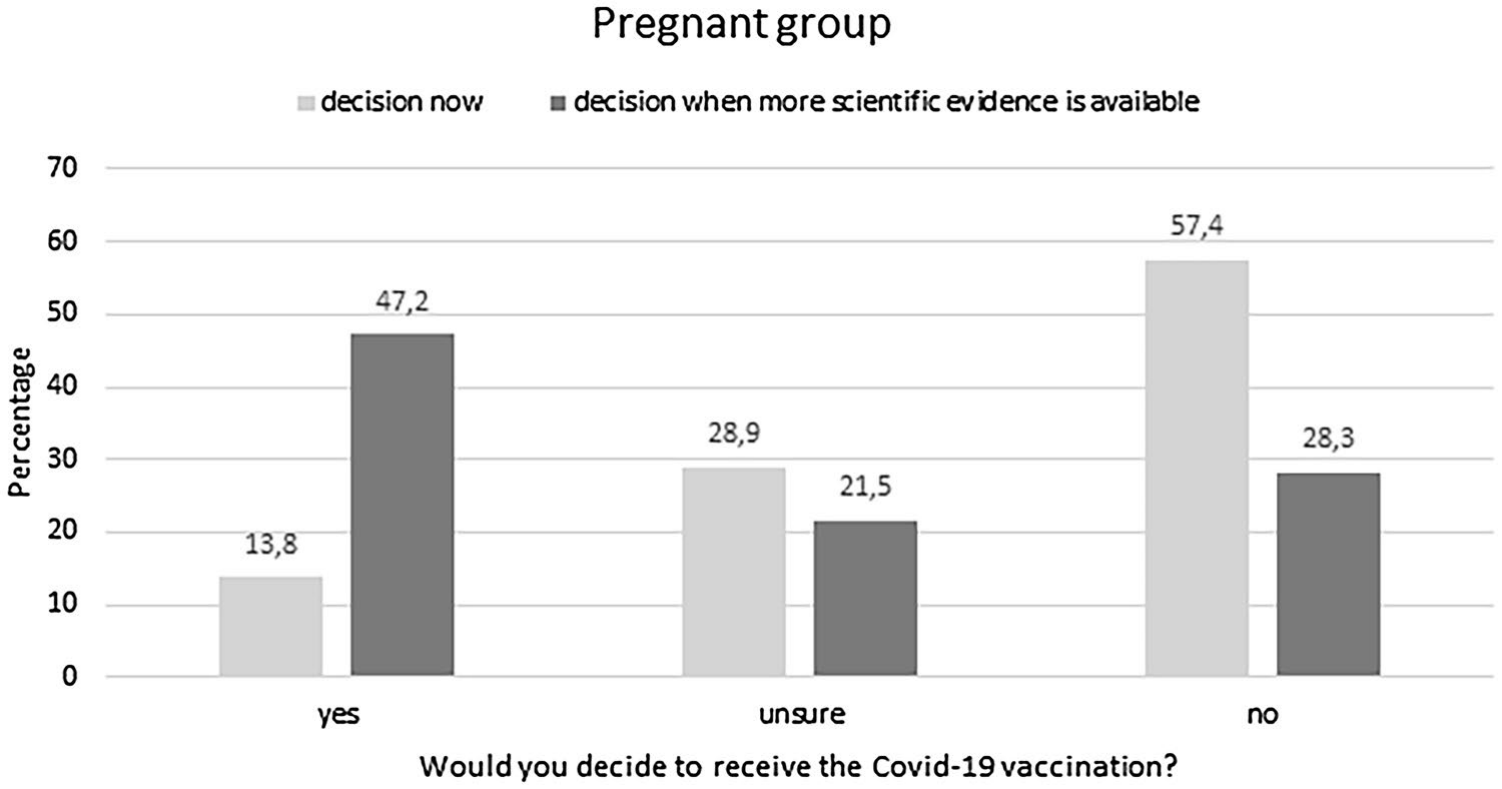
Willingness of the *pregnant group* to receive the COVID-19 vaccination. At the time of recruitment, the minority (13.8%) would decide to receive the COVID-19 vaccination. However, when asked whether they would be vaccinated if a scientific study would provide evidence for the safety of the vaccination 47.2% of the pregnant sample would decide to receive the vaccination

The non-vaccinated pregnant women (*n* = 1018) were asked whether they would take the vaccination if they would receive an offer for the COVID-19 vaccination. 140 (13.8%) would decide to get the vaccination, 294 (28.9%) are unsure and the majority (*n* = 584, 57.4%) do not want to be vaccinated (Fig. 2). Interestingly, this picture changed when the women were asked whether they would decide to be vaccinated if ongoing studies in the US and UK show that the vaccination is safe for pregnant women. Then 434 (47.2%) would decide to be vaccinated, 187 (21.5%) are unsure and 250 (28.7%) would decide against the vaccination (Fig. 2).

Of the 878 women, who indicated that they are unsure or not willing to be vaccinated, 695 (79.2%) stated that the reason was that they are pregnant, whereas the remaining 183 (20.8%) are generally against the COVID-19 vaccination. The most reported reasons (multiple-selection possible) for not wanting to be vaccinated during pregnancy were that the scientific data on the COVID-19 vaccination are too preliminary (*n* = 610, 87.8%), that they feel to have too little information (*n* = 423, 60.9%), they are anxious that the vaccine could harm their unborn (*n* = 597, 85.9%) or it could lead to complication with their pregnancy (*n* = 545, 78.4%).

Regarding the source of information, the *pregnant group* (*n* = 487) would want more information from, the most stated source were brochures and flyers (*N* = 364, 74.7%), followed by recommendations of professional societies (*n* = 348, 71.5%), on TV (*n* = 154, 31.6%) and in the newspaper (*n* = 145, 29.8%). Asking who would be the preferable contact person for questions regarding the COVID-19 vaccination of 159 women, who indicated to want a contact person, 140 (88.1%) like to talk to a gynecologist, 91 (57.2%) to a virologist and 82 (51.6%) to a midwife.

#### Breastfeeding group

In the *breastfeeding group,* 3.5% (*n* = 45) reported that they have been infected with SARS-CoV-2. Of these 45 women, 10 were pregnant when tested positive and 35 were breastfeeding. Most of them only had mild symptoms (77.8%).

In the *breastfeeding group,* 188 (13.7%) have received the COVID-19 vaccination (186 received the vaccination after birth and 2 during pregnancy). Of these 188 women, the majority (*n* = 107, 56.9%) received AstraZeneca, followed by BioNTech (*n* = 71) and Moderna (*n* = 9) and one woman did not remember. Reported side effects were a sore arm where the needle went in (*n* = 137, 73.7%), feeling unwell generally (*n* = 104, 55.9%), fever (*n* = 58, 31.2%) and chills (*n* = 71, 38.2%). The most common reasons for receiving the vaccination were because of their job (*n* = 147, 78.2%), they were more anxious to be infected than side effects from the vaccination (*n* = 117, 62.2%) and they want to protect their child (*n* = 141, 75.0%).

Of the non-vaccinated breastfeeding women (*n* = 1180), the majority (*n* = 465, 39.4%) would decide to get the vaccination if they receive an offer, 332 (28.1%) are unsure and 383, (32.5%) do not want to be vaccinated (Fig. 4).

Of the 715 women, who indicated that they are unsure or not willing to be vaccinated, 634 (88.7%) stated that the reason for this decision was that they are breastfeeding, whereas the remaining 81 (11.3%) are generally against the COVID-19 vaccinations. We then asked about more detailed reasons for not wanting to be vaccinated during the lactation period. Main reasons were that the scientific data on the COVID-19 vaccination are too preliminary (*n* = 390, 61.5%), too little information (*n* = 423, 60.9%) and concerns that the vaccine could harm their child (*n* = 433, 68.3%).

#### Group comparisons

**Fig. 3:**
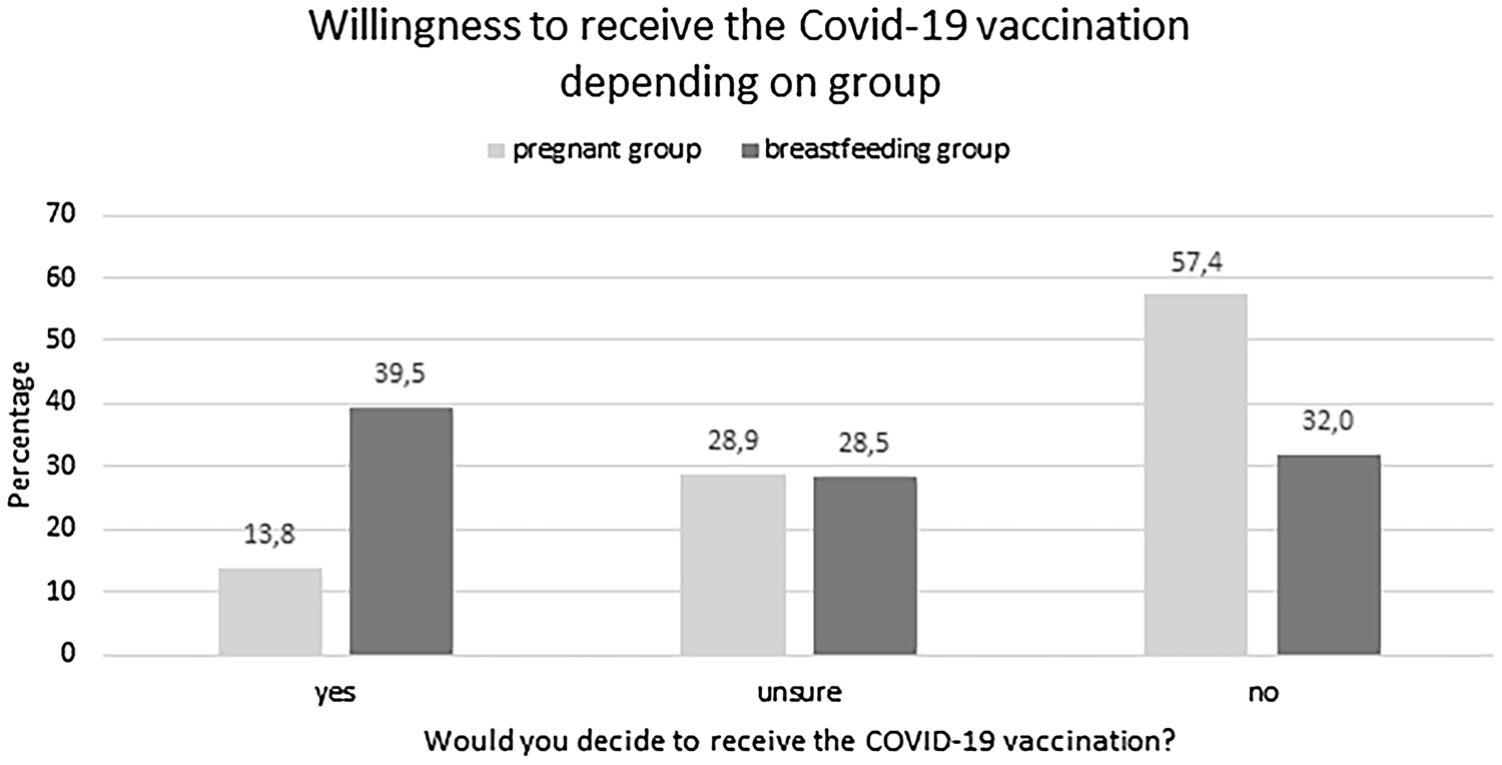
The willingness to receive the COVID-19 vaccinations significantly depends on the group. Less women in the pregnant group would decide to receive the vaccination that women in the breastfeeding group

Regarding the willingness to be vaccinated if they receive an offer for a COVID-19 vaccination, a chi-square-test revealed a significant effect of *group*, *χ*2 = 206.77, *p* < 0.001. Women in the *breastfeeding group* indicated more often than women in the *pregnant group* that they would decide to receive the vaccination (Fig. 3).

In both groups, women were more anxious to develop COVID-19 related symptoms than to be infected (*p* values < 0.001). In the pregnant group, the mean anxiety levels for symptoms was 57.12 ± 27.09 and for the infection 51.18 ± 25.55 and in the breastfeeding group the anxiety for symptoms was 55.14 ± 26.60 and for infection 48.06 ± 24.53. Independent-samples t-tests revealed that the two groups differed significantly regarding anxiety to be infected, *t*(2336) = 2.98, *p* = 0.003. The groups did not differ significantly regarding levels of anxiety for severe symptoms (*p* = 0.078).

**Fig. 4:**
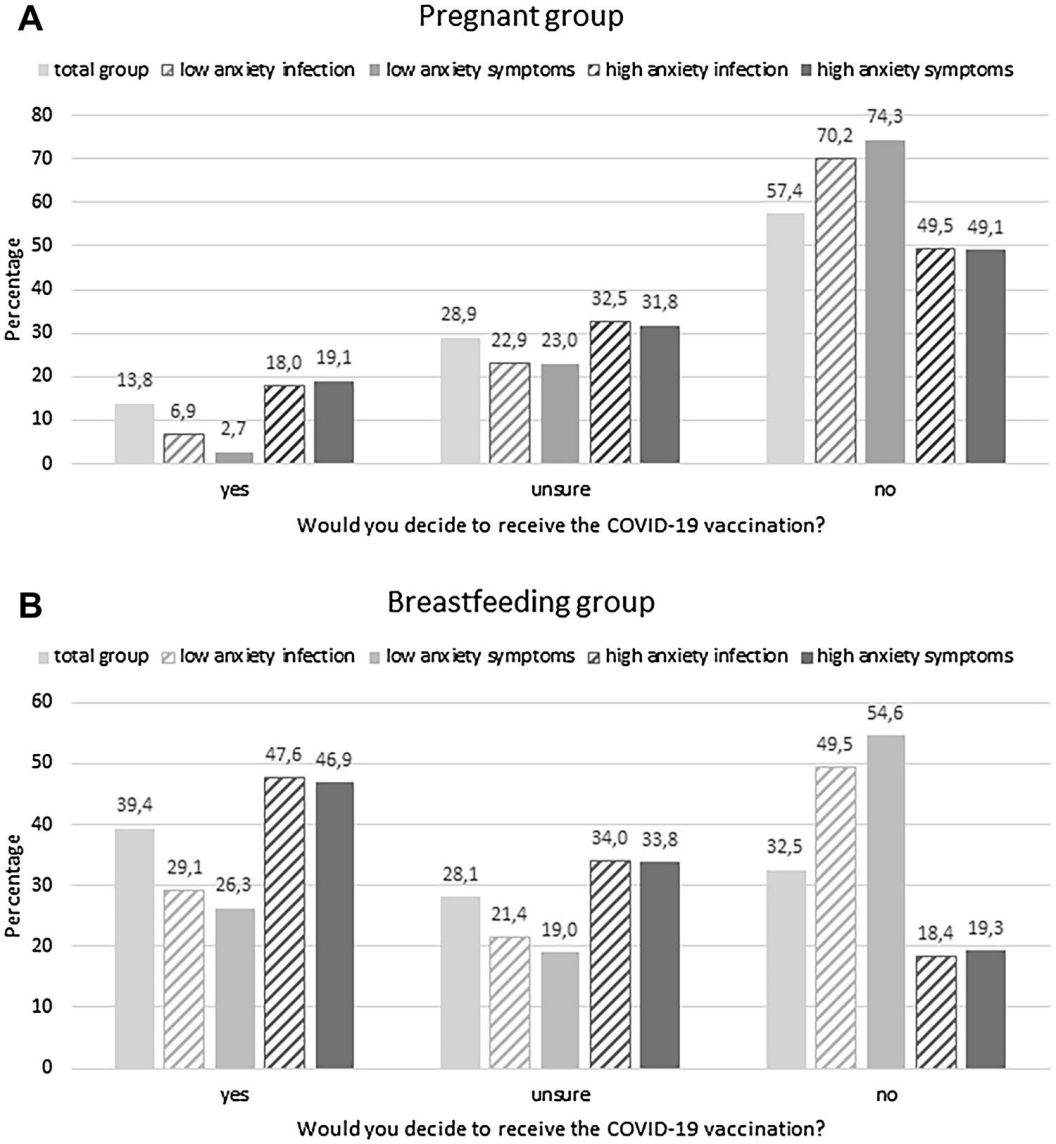
In both groups, the willingness to be vaccinated is dependent on whether women scored low or high on the visual analogue scales evaluating how anxious they are to be infected and to develop severe symptoms, respectively. In the pregnant group (**A**) and the breastfeeding group (**B**), the women in the high anxiety infection group and high anxiety symptoms group would decide to receive the COVID-19 vaccination more often than the low anxiety groups, whereas a higher percentage in the low anxiety groups would decide not to receive the vaccination than the high anxiety groups

Chi-squared-tests showed that anxiety to be infected (high vs. low) and anxiety of COVID-10 symptoms (high vs. low) is associated with the choice to be vaccinated (yes, no, unsure) in pregnant as well as breastfeeding women (all *p* values < 0.001, Fig. 4). Women with high anxiety levels regarding being infected and developing severe symptoms report higher percentages to want to be vaccinated against COVID-19.

A chi-square-test showed that in the *breastfeeding group* the willingness to be vaccinated is related to whether women are COVID-19 risk patients (*x*^*2*^ = 10.49, *p* = 0.005). The number of women, who would decide to receive the vaccine is higher in COVID-19 risk patients (47.3%) than in low-risk patients (37.5%). In the pregnant group the willingness was not related to the COVID-19 risk groups (*p* > 0.05).

## Discussion

### Main findings

The present study investigated perspectives of the COVID-19 vaccination of pregnant and breastfeeding women in Germany. The data show that especially during pregnancy the majority of women are not in favour of receiving the vaccine at the moment, which is in accordance with a study which was conducted in January 2021 among pregnant women in Italy [22]. Importantly, a much higher percentage would be in favour of receiving the vaccine if studies would show that the vaccination is safe for pregnant women, highlighting the importance of scientific studies including pregnant women, as they are at increased risk for a more severe course of the disease. In *the breastfeeding group,* the acceptance for receiving the vaccine is higher than in the *pregnant group*, however a large number of women is also unsure or not in favour of the vaccine. In both groups, the main reasons for not receiving the vaccine are that they feel to have too little information, scientific evidence regarding the safety is lacking and they are anxious that the vaccine could harm their offspring. Furthermore, the data show that the willingness to be vaccinated is related to the women’s anxiety regarding an infection and to develop disease symptoms in both groups. A greater anxiety regarding an infection and severe symptoms is associated with greater vaccine acceptance. Additionally, in the *breastfeeding group* the vaccine acceptance is higher if women are COVID-19 risk patients. Interestingly, in the *pregnant group,* the willingness to be vaccinated is not associated to risk factors for severe COVID-19. Especially this group of pregnant women was under individual considerations eligible for receiving the COVID-19 vaccination despite missing approval of the national STIKO recommendation. Therefore, the question must be made, if this restrictive institutional statement leads to restrictive information policies.

### Clinical implications

The here captured perspectives of pregnant and breastfeeding women are of high relevance and should be considered in future decisions regarding recommendations and information policy regarding COVID-19 vaccinations. It is eminent that women are anxious to receive the vaccination as they are afraid that the vaccine might harm the unborn or infant and could lead to pregnancy complications. Furthermore, a majority of women indicated that they do not want to receive the vaccination as they feel to have too little information. Here, it is important that the obstetric staff is informed about the most recent information regarding the COVID-19 vaccination as well as the current data regarding the risks a COVID-19 infection has for pregnant women. Especially, for women with risk factors such as higher age (> 35 years), higher body mass index, hypertension and further comorbidities [4, 5], it is important that the gynecologists discusses the benefits and risks of having the vaccine and that a joint decision is reached based on individual circumstances. Here, especially for breastfeeding women, the results demonstrate that women are in favor of receiving the vaccination if they are classified as COVID-19 risk patients based on their medical history. A good information policy is warranted to receive a high acceptance. Additionally, the data show that anxiety levels regarding a possible infection and developing symptoms influence the decision regarding the vaccination. In this respect, it is important that obstetric staff take reported anxiety levels seriously and try to inform and advise patients accordingly. Studies have revealed increased anxiety and levels of pregnant women during the pandemic [23–25], highlighting the psychological impact of the pandemic on this sensible group.

### Research implications

The here presented results emphasize the need of scientific studies which show the safety of the COVID-19 vaccination for pregnant and breastfeeding women to increase the acceptance of and trust in the vaccination. As the questionnaire revealed that the percentage of pregnant women who would decide to receive the COVID-19 vaccination raised to almost half of the pregnant women 47% (from 14% who would receive the vaccination now before scientific evidence), the need for trustworthy studies including pregnant women is highlighted. If studies show the safety for pregnant women, it can be hypothesised that the willingness of breastfeeding women will also increase.

It remains unclear to what extent potential complications of a COVID-19 infection in pregnancy, such as increased maternal morbidity and mortality as well as preterm birth [1, 2] could be reliably prevented by vaccinations. However, if studies show the safety of the vaccination, a benefit of the vaccination for valuable groups such as pregnant and breastfeeding women to also protect the unborn and infant, is expectable. In this respect, it would be desirable to repeat the survey when studies have been published on the safety of the vaccination for breastfeeding and lactating women and to investigate how the pattern changes regarding the acceptance of the vaccination in this special population.

### Strength and limitations

To the best of our knowledge, this is the largest study so far investigating perspectives of the COVID-19 vaccination of pregnant and breastfeeding women covering important aspects. The sample size as well as the demographics suggest that our data are representative for the German population. However, we do acknowledge as a limitation that the adaption of the present results to other countries may be limited, as the handling and distribution of the COVID-19 vaccination differs between countries. Nevertheless, we would argue that the results regarding the influence of anxiety levels on the willingness to be vaccinated as well as the presented importance of reliable studies showing that the vaccination is safe for pregnant women can be generalized. Another limitation which warrants a comment here, is that the applied questionnaire used self-created items to evaluate the attitudes towards the COVID-19 vaccination as well as the anxiety levels related to the vaccination and to be infected instead of validated questionnaires. However, as we were interested in specific anxiety levels regarding COVID-19 infections, no validated questionnaire exists so far.

## Conclusions

The present study highlights that, to increase the acceptance of pregnant and breastfeeding women regarding the COVID-19 vaccination, reliable scientific studies showing the safety of the vaccination in pregnancy, as well as more information on risks of a COVID-19 infection and benefits of the vaccination are warranted. It is important that healthcare professionals, especially gynecologists, discuss the benefits and risks of having the vaccine and reach a joint decision based on individual circumstances. This could help to protect pregnant and breastfeeding women as well as their offspring from a severe COVID-19 infection and might prevent adverse outcomes.

## Data Availability

The dataset used and analysed during the current study is available from the corresponding author on request.
